# Relative costs of offspring sex and offspring survival in a polygynous mammal

**DOI:** 10.1098/rsbl.2016.0417

**Published:** 2016-09

**Authors:** Hannah Froy, Craig A. Walling, Josephine M. Pemberton, Tim H. Clutton-Brock, Loeske E. B. Kruuk

**Affiliations:** 1 Institute of Evolutionary Biology, University of Edinburgh, Edinburgh, UK; 2 Department of Zoology, University of Cambridge, Cambridge, UK; 3 Research School of Biology, Australian National University, Canberra, Australian Capital Territory, Australia

**Keywords:** cost of reproduction, *Cervus elaphus*, sex allocation, wild ungulate population

## Abstract

Costs of reproduction are expected to be ubiquitous in wild animal populations and understanding the drivers of variation in these costs is an important aspect of life-history evolution theory. We use a 43 year dataset from a wild population of red deer to examine the relative importance of two factors that influence the costs of reproduction to mothers, and to test whether these costs vary with changing ecological conditions. Like previous studies, our analyses indicate fitness costs of lactation: mothers whose calves survived the summer subsequently showed lower survival and fecundity than those whose calves died soon after birth, accounting for 5% and 14% of the variation in mothers' survival and fecundity, respectively. The production of a male calf depressed maternal survival and fecundity more than production of a female, but accounted for less than 1% of the variation in either fitness component. There was no evidence for any change in the effect of calf survival or sex with increasing population density.

## 1. Introduction

An understanding of the costs of reproduction is fundamental to life-history evolution theory [1]. The energetic costs of raising offspring increase as they progress through the period of parental investment, generating fitness costs for the parents [2]. These costs can vary with the characteristics of both the parents (such as their age [3] or social dominance [4]) and the offspring (such as their size or sex [5]). For example, in sexually size-dimorphic species, offspring of the larger sex commonly require more resources, and producing and rearing them can depress mothers' subsequent survival or breeding success, with implications for sex ratio evolution [4,6,7]. Reproductive costs may be ecologically or physiologically mediated [8] and may also vary with environmental conditions [9,10] though we know little about the relative magnitude of these effects.

Long-term, individual-based studies provide an excellent opportunity to explore the costs of reproduction in wild animals [3,9,10]. Here, we extend earlier work on red deer on the Isle of Rum which has shown that reproduction generates substantial costs to mothers' subsequent survival and fecundity, which vary with both the longevity of the offspring and its sex [4,5,11]. We add an additional 26 years' data and use novel statistical methods to quantify the relative magnitude of costs to maternal survival and fecundity. In addition, we investigate whether the costs of rearing offspring and the relative costs of producing sons and daughters changed with population density, which increased over the study period [12].

## 2. Material and methods

The unmanaged population of red deer in the North Block of the Isle of Rum, Scotland, has been studied since 1971, with survival and reproductive history of individuals known from regular censuses [4,11]. Females that conceive during the autumn rut give birth to a single calf in May–June, and approximately 10% of calves die in their first two weeks of life. Winter mortality affecting all ages occurs January–March. We used data on all females (aged 3–18 years) that gave birth to a calf of known sex from 1971–2013 inclusive [13].

We examined the effects of calf survival and calf sex in a given year on the subsequent survival and fecundity of the mother. Maternal survival was assessed as survival to 1 May the following year (*n* = 2888 observations of 636 females), and fecundity by whether she gave birth to a calf the following year, conditional on her survival (*n* = 2600 observations of 602 females). Calf sex and calf survival to 1 October of the year they were born were included in the models as 2-level fixed factors. Maternal age (linear and quadratic), population size (over the subsequent winter; see the electronic supplementary material) and calf birth date (days since 1 May) were included as fixed covariates. Year was fitted as a random multi-level factor in both models, and maternal identity was fitted as a random term in the fecundity model (see the electronic supplementary material). We tested for differential costs of male and female calves depending on whether they survived to 1 October by including an interaction between calf sex and calf survival in each model, and for changes in costs across varying population densities by including an interaction between either variable and population size.

Maternal survival and fecundity were modelled as binary traits in Bayesian generalized linear mixed models (GLMMs) with the R package MCMCglmm [14], using the categorical family and logit link function. Continuous predictor variables were mean centred prior to inclusion in models. Parameter estimates are presented as the posterior mode with 95% credible intervals of 2000 samples with minimal autocorrelation (iterations: 1.1 × 10^6^; burn-in: 1 × 10^5^; thinning interval: 500). Marginal *R*^2^ indicates the percentage of variance explained by the fixed effect component of a model, and can be estimated for GLMMs as the variance of the fixed effects divided by the total variance (×100), calculated on the link scale [15]. We used the change in marginal *R*^2^ (Δ*R*^2^) when each fixed effect was dropped from the model in turn to estimate the variance in maternal survival and fecundity explained by each of the fixed effects.

## 3. Results

**Figure 1.**
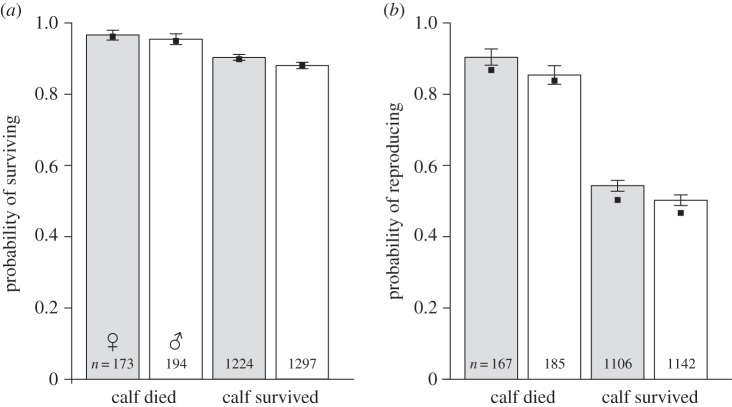
Effects of calf sex and calf survival on (*a*) maternal survival and (*b*) fecundity the year after giving birth. Bars show raw data with standard errors; filled bars represent female calves, and unfilled bars represent males. Black squares show predictions from models incorporating other variables.

Mothers of calves that survived to 1 October were less likely to survive the next winter (figure 1*a*; *P*_MCMC_ < 0.001) or to breed again the following year (figure 1*b*; *P*_MCMC_ < 0.001) than mothers whose calves died during the course of the summer; this cost explained a substantial proportion of variation in maternal survival and fecundity (Δ*R*^2^ = 5% and 14%, respectively; table 1). Mothers that gave birth to a male calf were also less likely to survive to the following year (figure 1*a*; *P*_MCMC_ = 0.023), and less likely to give birth the following spring if they did survive (figure 1*b*; *P*_MCMC_ = 0.003). However, calf sex explained less than 1% of the variation both in survival probability and fecundity (table 1). There was no significant interaction between the effects of calf sex and calf survival on maternal survival or fecundity (electronic supplementary material, table S1). In addition, there was no evidence for a significant interaction between population size and the effect of either calf survival or calf sex on either maternal survival or fecundity (electronic supplementary material, table S1). For a summary of other fixed effects, see the electronic supplementary material.

**Table 1.** Summary of fixed and random effects from generalized linear mixed models (GLMMs) of maternal survival and fecundity the year after giving birth to a calf. (‘Parameter estimate’ gives the mode of the posterior distribution for the coefficient of that variable; parameter estimates are on the link scale for the GLMM (logit link for binomial errors). Δ*R*^2^ shows the change in marginal *R*^2^ (which is a %) when each fixed effect is dropped from the model in turn.)

	variable	parameter estimate	lower CI	upper CI	*P*_MCMC_	Δ*R*^2^
survival	*n* = 2888 (636 females)				marginal *R*^2^ = 27.64%	
random effects	year	0.638	0.267	1.099		
fixed effects	age	0.421	0.202	0.666	<0.001	−16.66
	age^2^	−0.038	−0.051	−0.027	<0.001	
	population size	−0.014	−0.025	−0.003	0.004	−2.79
	calf birth date	−0.021	−0.030	−0.014	<0.001	−2.21
	calf sex: male	−0.384	−0.705	−0.080	0.023	−0.65
	calf survival	−1.844	−2.582	−1.198	<0.001	−5.03
fecundity	*n* = 2600 (602 females)				marginal *R*^2^ = 29.04%	
random effects	year	0.754	0.348	1.172		
	maternal ID	1.963	1.381	2.612		
fixed effects	age	0.901	0.697	1.088	<0.001	−3.55
	age^2^	−0.054	−0.065	−0.043	<0.001	
	population size	−0.029	−0.040	−0.019	<0.001	−7.19
	calf birth date	−0.043	−0.052	−0.034	<0.001	−5.55
	calf sex: male	−0.347	−0.570	−0.085	0.003	−0.50
	calf survival	−3.564	−4.077	−3.071	<0.001	−14.38

## 4. Discussion

Successful reproduction was costly for red deer females in terms of future survival and fecundity (figure 1 and table 1). Whether or not the calf was alive at the onset of autumn was the greatest determinant of these post-parturition costs, with mothers of calves that survived to 1 October being 6.5% less likely to survive the winter and 36.7% less likely to give birth the following year. This result supports previous findings from this study population and is presumably a consequence of the substantial energetic costs of lactation [11,16]. More than 85% of calves that die in summer die within two weeks of birth, meaning their mothers experience minimal costs of lactation. If calves survive to 1 October, they usually survive the next few months (80% of winter deaths occur in February–April), meaning their mothers bear the full costs of lactation, potentially lactating through the winter months if they fail to conceive again [16].

Producing male calves was more costly than producing female calves (figure 1) [4,5]. Our analysis shows that the effect of calf sex was small in comparison with that of calf survival, explaining less than 1% of the variation in subsequent maternal survival and fecundity (table 1). Previous work suggests that the additional costs of raising sons are greater for subordinate mothers than for dominants so that the relative costs of raising sons may vary in relation to the mother's phenotype [4]. We found no evidence of any interaction between the effects of calf sex and calf survival, suggesting that the relative cost of males was the same regardless of how long they lived. The same was true if we considered only whether the calf survived beyond its first two weeks, so the relative cost of males did not increase even if their mothers experienced the main period of lactation (from birth to three months). One possible interpretation of these results is that the difference in cost of male versus female offspring is generated during gestation (male calves are approx. 5.5% heavier at birth). However, evidence that costs of gestation are small in comparison with those of lactation [11] and that sons suck more than daughters [5] suggest that this is unlikely; the lack of interaction may therefore reflect a lack of statistical power given the small magnitude of the main effect of calf sex.

Our analyses showed strong associations with calf birth date: mothers of early-born calves were more likely to survive and to give birth the following year (explaining 2% and 5% of the variance, respectively; table 1). This effect could be driven by differences in female condition, since mothers in good condition are likely to conceive and give birth earlier, and also have higher future survival and fecundity. Further analysis revealed that the effect of calf birth date was dependent on calf survival, only being significant when the calf survived beyond 1 October (electronic supplementary material, table S2), which suggests that mothers in poor condition (who give birth later) suffer higher costs of successful reproduction, as has also been observed in other ungulates [17].

We found evidence of density-dependence in maternal survival and fecundity (table 1). However, although the effect of calf sex on fecundity was non-significant in the most recent 26 years of data added for this analysis, when population density was high (−0.223 [−0.494− 0.085], *P*_MCMC_ = 0.137; electronic supplementary material, table S3), there is no indication that the costs associated with calf survival or sex varied with increasing density (electronic supplementary material, table S1). A possible explanation of this effect is that maternal investment in lactation is adjusted to the mother's food intake so that variation in food availability affects the growth and survival of calves rather than the survival and fecundity of their mothers [18].

Several other studies of sexually dimorphic mammals have shown that mothers invest more energy in sons than daughters [19] but relatively few have been able to investigate whether this affects maternal fecundity [9] or survival [20]. Our ability to detect survival costs is unusual for such a long-lived species [18], and may in part be attributable to the large sample sizes available across many years. It may also reflect the relatively harsh conditions on Rum, which are likely to accentuate the reproductive costs of energetic investment [9,10]. We detected reproductive costs despite extensive female heterogeneity, which can frequently mask costs in the highest quality individuals [17,21]. However, it is likely that we are underestimating the total costs of reproduction, because individuals are expected to reduce energetic investment in breeding to minimize fitness costs [18,22].

In summary, we found considerable costs of successful reproduction for female red deer in terms of future survival and fecundity. We found evidence for a significantly higher cost of sons than daughters, although this difference was smaller than the effects of juvenile survival. Despite density-dependence in both aspects of maternal performance, we found no indication that reproductive costs varied with ecological conditions. Our analyses illustrate the value of long-term datasets in affording tests of the generality of life-history patterns across changing environments.

## Supplementary Material

Further methods and analyses of Relative Costs of Offspring Sex and Offspring Survival in a Polygynous Mammal

## Data accessibility

Data are available in the Dryad Digital Repository: http://dx.doi.org/10.5061/dryad.6rj63 [23].
