# Supplementary material for: Relative costs of offspring sex and offspring survival in a polygynous mammal
Source: Biol Lett. 2016 Sep;12(9):20160417. doi: 10.1098/rsbl.2016.0417 (PMC5046923; doi:10.1098/rsbl.2016.0417)
Supplement: Further methods and analyses of Relative Costs of Offspring Sex and Offspring Survival in a Polygynous Mammal [file rsbl20160417supp1.docx]

**Supplementary materials: Relative costs of offspring sex and offspring survival in a polygynous mammal**

Hannah Froy, Craig A Walling, Josephine M Pemberton, Tim H Clutton-Brock, Loeske EB Kruuk,

Additional methodological details

Population size was estimated as the number of adult females seen in >10% of censuses in January – May of the winter following the birth of the calf. Population density in the study area has increased over the course of the study, with effects on a variety of life-history traits (1). Mothers with unknown year of death were excluded from the analyses, as were the final observations of mothers who were shot (when roaming outside of the study area). Maternal ID was included as a multi-level random factor in the fecundity model, but not in the survival model because model convergence was poor when this term was included (high autocorrelation among stored samples of the MCMC chain, despite sampling every 500 iterations). This is likely because of the limited variance in survival between individuals, the only variation between females being the number of years survived (1s) prior to their death (a single 0).

Effects of other variables in the main model

Calf birth date explained 2% and 5% of variation in subsequent maternal survival and fecundity respectively, with mothers of later-born calves less likely to survive and give birth the following year. Over-winter survival varied with age (explaining 17% of variation; Table 1) and showed a senescent decline in later life (2, 3). Similarly, the probability of giving birth in consecutive years showed a quadratic relationship with maternal age, being highest among prime-age females (4). There was also a negative correlation between population density and the probability of surviving and giving birth in consecutive years (explaining 3% and 7% of variation, respectively; Table 1), indicating a classic density-dependent response (5, 6).

Additional statistical analyses

To test whether the negative effect of calf birth date reflected a consistent reduction in survival and fecundity for mothers of late-born calves, or whether this indicated a higher cost of successful reproduction for these females in poor condition, we included an interaction between calf birth date and calf survival; shown in Table S2.

Our analyses differed from those presented in earlier publications (7, 8) both in terms of the dataset used and the statistical methods. To determine whether any differences in conclusions were due to the different dataset versus different methods, we also ran the analyses for the dataset used in previous papers (1971–1987) and then for the new data added for this analysis (1988–2013) separately; these results are presented in Table S3.

Results of additional statistical analyses

There was a significant interaction between calf birth date and calf survival (Table S2), which indicated that that the effect of calf birth date was only important if the calf survived the summer (see Main Text for further discussion).

The effect of calf sex on maternal survival was consistent across the study period, but the negative effect of male calves on fecundity was not, being significant in the early dataset, but reduced and no longer significant in the most recent 26 years (Table S3). However, this difference in the effect of calf sex between the two periods was not statistically significant (interaction between calf sex and study period: -0.383 [-0.921 – 0.137], P_MCMC_=0.172).

Table S1. The effect of two-way interactions between calf sex, calf survival and population size on maternal survival and fecundity the year after giving birth to a calf. Interactions were each added in turn to the main binomial generalised linear mixed models (Table 1). ‘Parameter estimate’ gives the mode of the posterior distribution for each interaction, on the logit link scale. None of the interactions were significant.

|  | **Interaction** | **Parameter estimate** | **Lower CI** | **Upper CI** | **P_MCMC_** |
| --- | --- | --- | --- | --- | --- |
| **Survival** | n=2888 (636 females) | |  |  |  |
|  | Calf survival * Calf sex | 0.094 | -1.239 | 1.552 | 0.907 |
|  | Calf sex * Population size | -0.006 | -0.019 | 0.007 | 0.354 |
|  | Calf survival * Population size | -0.009 | -0.034 | 0.018 | 0.512 |
|  |  |  |  |  |  |
| **Fecundity** | n=2600 (602 females) | |  |  |  |
|  | Calf survival * Calf sex | 0.490 | -0.445 | 1.471 | 0.328 |
|  | Calf sex * Population size | 0.004 | -0.005 | 0.014 | 0.480 |
|  | Calf survival * Population size | -0.018 | -0.037 | <0.001 | 0.066 |

Table S2. Summary of fixed and random effects from binomial generalised linear mixed models of maternal survival and fecundity the year after giving birth to a calf, including an additional interaction between calf survival and calf birth date. ‘Parameter estimate’ gives the mode of the posterior distribution for the coefficient of that variable, on the logit link scale.

|  | **Variable** | **Parameter estimate** | **Lower CI** | **Upper CI** | **P_MCMC_** |
| --- | --- | --- | --- | --- | --- |
| **Survival** | n=2888 (636 females) | |  |  |  |
| Random effects | Year | 0.637 | 0.278 | 1.099 |  |
| Fixed effects | Age | 0.434 | 0.197 | 0.648 | <0.001 |
|  | Age^2^ | -0.039 | -0.050 | -0.027 | <0.001 |
|  | Population size | -0.014 | -0.025 | -0.003 | 0.006 |
|  | Calf sex: Male | -0.395 | -0.740 | -0.092 | 0.019 |
|  | Calf survival | -1.704 | -2.478 | -1.080 | <0.001 |
|  | Calf birth date | 0.012 | -0.023 | 0.047 | 0.531 |
|  | Calf Survival * Calf birth date | -0.037 | -0.073 | >-0.001 | 0.032 |
|  |  |  |  |  |  |
| **Fecundity** | n=2600 (602 females) | |  |  |  |
| Random effects | Year | 0.784 | 0.383 | 1.258 |  |
|  | Maternal ID | 2.070 | 1.437 | 2.726 |  |
| Fixed effects | Age | 0.918 | 0.724 | 1.131 | <0.001 |
|  | Age^2^ | -0.054 | -0.066 | -0.042 | <0.001 |
|  | Population size | -0.030 | -0.041 | -0.021 | <0.001 |
|  | Calf sex: Male | -0.349 | -0.604 | -0.095 | 0.007 |
|  | Calf survival | -3.411 | -3.920 | -2.894 | <0.001 |
|  | Calf birth date | -0.016 | -0.032 | >-0.001 | 0.062 |
|  | Calf survival * Calf birth date | -0.038 | -0.059 | -0.020 | <0.001 |

Table S3. Summary of fixed and random effects from binomial generalised linear mixed models of maternal survival and fecundity the year after giving birth to a calf, split into the two periods of 1971–1987 (as presented in previous studies (6-9)) and 1988–2013. ‘Parameter estimate’ gives the mode of the posterior distribution for the coefficient of that variable, on the logit link scale.

|  | **Variable** | **Parameter estimate** | | **Lower CI** | **Upper CI** | **P_MCMC_** |
| --- | --- | --- | --- | --- | --- | --- |
| **Survival 1971**–**1987** | n=935 (221 females) | | |  | marginal R^2^=36.20% | |
| Random effects | Year | | 0.505 | <0.001 | 1.383 |  |
| Fixed effects | Age | | 0.441 | 0.017 | 0.893 | 0.051 |
|  | Age^2^ | | -0.038 | -0.062 | -0.016 | <0.001 |
|  | Population size | | -0.023 | -0.039 | -0.008 | 0.007 |
|  | Calf birth date | | -0.027 | -0.042 | -0.013 | <0.001 |
|  | Calf sex: Male | | -0.482 | -1.060 | -0.099 | 0.093 |
|  | Calf survival | | -2.707 | -4.347 | -1.206 | <0.001 |
|  |  | |  |  |  |  |
| **Survival 1988**–**2013** | n=1953 (502 females) | | |  | marginal R^2^=26.57% | |
| Random effects | Year | | 0.789 | 0.259 | 1.480 |  |
| Fixed effects | Age | | 0.406 | 0.130 | 0.690 | <0.001 |
|  | Age^2^ | | -0.039 | -0.052 | -0.024 | <0.001 |
|  | Population size | | -0.009 | -0.034 | 0.015 | 0.440 |
|  | Calf birth date | | -0.019 | -0.028 | -0.009 | <0.001 |
|  | Calf sex: Male | | -0.334 | -0.730 | -0.037 | 0.083 |
|  | Calf survival | | -1.655 | -2.485 | -0.921 | <0.001 |
|  |  | |  |  |  |  |
| **Fecundity 1971**–**1987** | n=850 (209 females) | | |  | marginal R^2^=36.09% | |
| Random effects | Year | | 0.979 | 0.205 | 2.163 |  |
|  | Maternal ID | | 2.325 | 1.133 | 3.630 |  |
| Fixed effects | Age | | 1.164 | 0.771 | 1.544 | <0.001 |
|  | Age^2^ | | -0.065 | -0.086 | -0.042 | <0.001 |
|  | Population size | | -0.035 | -0.054 | -0.020 | 0.001 |
|  | Calf birth date | | -0.033 | -0.048 | -0.020 | <0.001 |
|  | Calf sex: Male | | -0.646 | -1.101 | -0.175 | 0.005 |
|  | Calf survival | | -4.107 | -5.191 | -3.132 | <0.001 |
|  |  | |  |  |  |  |
| **Fecundity 1988**–**2013** | n=1750 (462 females) | | |  | marginal R^2^=26.40% | |
| Random effects | Year | | 0.822 | 0.307 | 1.479 |  |
|  | Maternal ID | | 1.867 | 1.161 | 2.652 |  |
| Fixed effects | Age | | 0.787 | 0.544 | 1.027 | <0.001 |
|  | Age^2^ | | -0.048 | -0.063 | -0.034 | <0.001 |
|  | Population size | | -0.038 | -0.061 | -0.015 | 0.003 |
|  | Calf birth date | | -0.049 | -0.061 | -0.039 | <0.001 |
|  | Calf sex: Male | | -0.223 | -0.494 | 0.085 | 0.137 |
|  | Calf survival | | -3.381 | -3.998 | -2.784 | <0.001 |

1. Stopher KV, Bento AI, Clutton-Brock TH, Pemberton JM, Kruuk LE. Multiple pathways mediate the effects of climate change on maternal reproductive traits in a red deer population. Ecology. 2014;95(11):3124-38.

2. Nussey DH, Kruuk LE, Morris A, Clutton-Brock TH. Environmental conditions in early life influence ageing rates in a wild population of red deer. Current Biology. 2007;17(23):R1000-R1.

3. Catchpole EA, Fan Y, Morgan BJT, Clutton-Brock TH, Coulson T. Sexual dimorphism, survival and dispersal in red deer. Journal of Agricultural, Biological, and Environmental Statistics. 2004;9(1):1-26.

4. Nussey DH, Kruuk LE, Morris A, Clements MN, Pemberton JM, Clutton‐Brock TH. Inter‐and intrasexual variation in aging patterns across reproductive traits in a wild red deer population. The American Naturalist. 2009;174(3):342-57.

5. Clutton-Brock TH, Albon SD, Guinness FE. Parental investment and sex differences in juvenile mortality in birds and mammals. Nature. 1985;313(5998):131-3.

6. Clutton-Brock TH, Guinness FE, Albon SD. The costs of reproduction to red deer hinds. Journal of Animal Ecology. 1983;52(2):367-83.

7. Clutton-Brock TH, Albon SD, Guinness FE. Fitness costs of gestation and lactation in wild mammals. Nature. 1989;337(6204):260-2.

8. Gomendio M, Clutton-Brock TH, Albon SD, Guinness FE, Simpson MJ. Mammalian sex ratios and variation in costs of rearing sons and daughters. Nature. 1990;343(6255):261-3.
